# HbA1C and Cancer Risk in Patients with Type 2 Diabetes – A Nationwide Population-Based Prospective Cohort Study in Sweden

**DOI:** 10.1371/journal.pone.0038784

**Published:** 2012-06-14

**Authors:** Junmei Miao Jonasson, Jan Cederholm, Björn Eliasson, Björn Zethelius, Katarina Eeg-Olofsson, Soffia Gudbjörnsdottir

**Affiliations:** 1 Center of Registers in Region Västra Götaland, Gothenburg, Sweden; 2 Department of Oncology, Institute of Clinical Sciences, Sahlgrenska Academy, University of Gothenburg, Gothenburg, Sweden; 3 Department of Public Health and Caring Sciences/Family Medicine and Clinical Epidemiology, Uppsala University, Sweden; 4 Department of Medicine, Sahlgrenska University Hospital, University of Gothenburg, Göteborg, Sweden; 5 Department of Public Health and Caring Sciences/Geriatrics, Uppsala University, Uppsala, Sweden; 6 Medical Products Agency, Uppsala, Sweden; Fundación para la Prevención y el Control de las Enfermedades Crónicas No Transmisibles en América Latina (FunPRECAL), Argentina

## Abstract

**Background:**

Diabetes is associated with increased cancer risk. The underlying mechanisms remain unclear. Hyperglycemia might be one risk factor. HbA1c is an indicator of the blood glucose level over the latest 1 to 3 months. This study aimed to investigate association between HbA1c level and cancer risks in patients with type 2 diabetes based on real life situations.

**Methods:**

This is a cohort study on 25,476 patients with type 2 diabetes registered in the Swedish National Diabetes Register from 1997–1999 and followed until 2009. Follow-up for cancer was accomplished through register linkage. We calculated incidences of and hazard ratios (HR) for cancer in groups categorized by HbA1c ≤58 mmol/mol (7.5%) versus >58 mmol/mol, by quartiles of HbA1c, and by HbA1c continuously at Cox regression, with covariance adjustment for age, sex, diabetes duration, smoking and insulin treatment, or adjusting with a propensity score.

**Results:**

Comparing HbA1c >58 mmol/mol with ≤58 mmol/mol, adjusted HR for all cancer was 1.02 [95% CI 0.95–1.10] using baseline HbA1c, and 1.04 [95% CI 0.97–1.12] using updated mean HbA1c, and HRs were all non-significant for specific cancers of gastrointestinal, kidney and urinary organs, respiratory organs, female genital organs, breast or prostate. Similarly, no increased risks of all cancer or the specific types of cancer were found with higher quartiles of baseline or updated mean HbA1c, compared to the lowest quartile. HR for all cancer was 1.01 [0.98–1.04] per 1%-unit increase in HbA1c used as a continuous variable, with non-significant HRs also for the specific types of cancer per unit increase in HbA1c.

**Conclusions:**

In this study there were no associations between HbA1c and risks for all cancers or specific types of cancer in patients with type 2 diabetes.

## Introduction

Type 2 diabetes has been associated with increased risk of several cancer types [1], such as breast cancer and gastrointestinal cancer, and a decreased risk of prostate cancer has also been found. The underlying mechanisms have been debated and remain unclear [1], [2]. A consensus statement from the societies of diabetes and cancer researchers and experts has recently been published [1], [3]. This statement points out that there are unanswered questions concerning the observed associations between diabetes and cancer. We do not know the role of diabetes itself or the typical metabolic disturbances in diabetes, or shared risk factors of diabetes and cancer, or the diabetes medications in relation to the observed links between cancer and diabetes [1]–[4]. Hyperglycemia, one of the main characteristics of diabetes, is considered one possible reason for increased risk of cancer in diabetes [5]. HbA1c is a test that measures the amount of glycated hemoglobin in blood, and gives a stable estimate of blood glucose control over the last 1 to 3 months [6].

More intensive glucose control in patients with type 2 diabetes did not affect the risk of cancer incidence [7] or mortality [7], [8] in two randomized trials with mean of 3.5 or 5 years of follow-up. Similar results were shown in a meta-analysis of major trials [9]. Inconsistent results were reported from previous observational studies on the relationship between HbA1c levels and cancer incidence or mortality, for reasons of different study cohorts, i.e., ‘apparently healthy people’, mixed group of people with or without diabetes, or patients without aclearly defined type of diabetes[10]–[13]. We therefore performed a nationwide population-based observational study based on Swedish patient registers to assess the associations between HbA1c and incidence of all cancers or cancers of specific types in patients with type 2 diabetes.

## Methods

**Figure 1:**
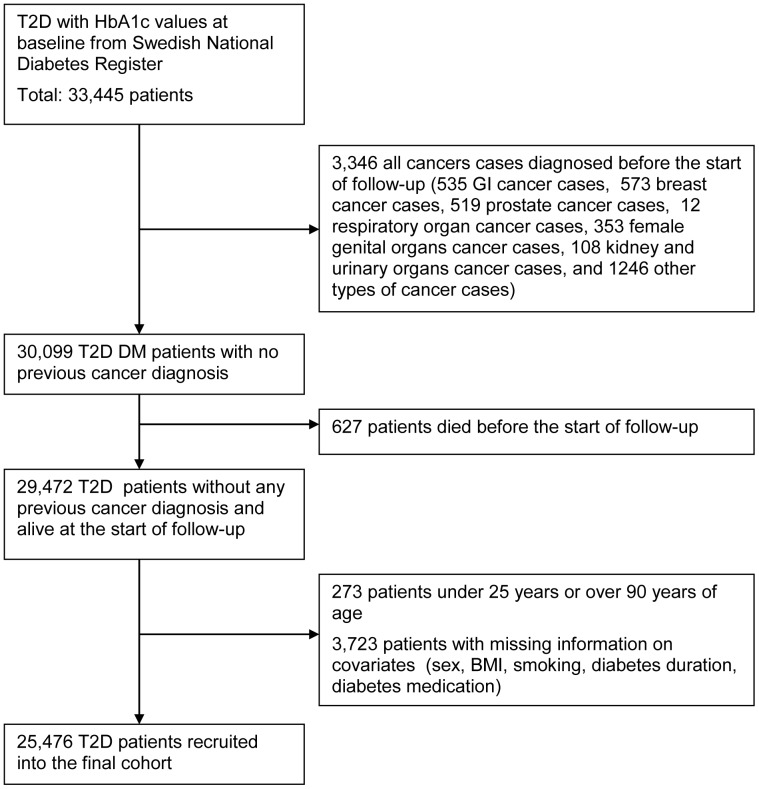
The flow chart presenting the compilation of the study cohort.

This is a prospective cohort study based on Swedish Registers: the National Diabetes Register, the Cancer Register and the Causes of Death Register. More details about these registers have been described in a previous publication [14]. The study cohort, all patients with type 2 diabetes, was selected from the Swedish National Diabetes Register, with baseline years 1997–1999. The cohort selected for the current study is based upon registry entries in the Swedish National Diabetes Register from 1997–1999, which are completely different from those used for the study on insulin glargine and cancer risk, where the cohort was selected based on registry entries in the Swedish Prescribed Drug Register in 2005 [14]. Outcomes during follow-up of the study cohort were obtained through linkage to the Cancer Register and the Causes of Death Register, with use of the Swedish personal identity number, a unique identifier assigned to every resident in Sweden and allowing linkage between different registers [14]. Figure 1 presents the compilation of the study cohort in the form of a flow chart.

### Study Cohort

The study cohort consisted of 25,476 type 2 diabetes patients aged 25–90 years, with baseline data in 1997–1999 available for all analyzed variables. All included patients have agreed by informed consent to register in the NDR before inclusion. The definition of type 2 diabetes is treatment with diet only, oral hypoglycaemic agents only, or onset age of diabetes >40 years combined with insulin only or insulin and oral agents. Exclusion criteria were diagnosis of cancer of study interest or death before the start of follow-up, as obtained through linkage to the Cancer Register and the Causes of Death Register.

### Exposure

HbA1c analyzes were quality assured nationwide by regular calibration with the HPLC Mono-S method, and HbA1c values were converted to the DCCT standard using the formula: HbA1c (DCCT)  = 0.923×HbA1c (Mono-S) +1.345; R2 = 0.998 [15]. HbA1c was measured at baseline. HbA1c was also measured over the follow-up period as an updated mean of annual measurements, with the last observation carried forward for missing data. HbA1c values were used annually until an event, or until censor date in case of no event.

### Follow-up

Cohort members were followed from the first day of the year after the baseline clinical examinations in 1997–1999 until the first diagnosis of outcome, or death, or the end of follow-up, December 31, 2009.

### Outcomes

Study outcomes were the first diagnosis of any malignant cancer (all cancer), or the first diagnosis of a specific type of malignant cancer during follow-up. The outcomes were identified from the Cancer Register using ICD coding. First incident all cancer was defined as ICD-10 codes (C00–C97, D00–D09, D37–D48) (International Classification of Diseases, 10th revision), and the following specific types of cancer were also investigated: first incident gastrointestinal cancer (ICD-10 code C15–C25), first incident breast cancer in women (C50), and first incident prostate cancer in men (C61). For cancer of specific sites, we included only tumors that were histopathologically classified as adenocarcinoma (WHO/HS/CANC/24.1 histology code 096). We also investigated first incident cancer of kidney and urinary organs (C64–C68), respiratory organs (C30–C39) and female genital organs (C51–C58).

### Potential Confounding Factors

Age, sex, diabetes duration, baseline body mass index [BMI (kg/m^2^)], smoking and insulin treatment were regarded as potential confounding factors. BMI was calculated as weight divided by height squared. A smoker was defined as a patient who smoked one or more cigarettes per day, or who smoked tobacco using a pipe, or who had stopped smoking within the past three months.

### Statistical Methods

The cohort was divided according to its median HbA1c value into two groups of baseline, HbA1c ≤58 mmol/mol (7.5%) or >58 mmol/mol, and into two groups of updated mean, HbA1c ≤58 mmol/mol or >58 mmol/mol. Baseline characteristics are presented in Table 1 as mean values with one standard deviation (SD) and frequencies (%) in each group of baseline or updated mean HbA1c ≤58 mmol/mol, or baseline or updated mean HbA1c >58 mmol/mol. Significance test between the groups were conducted with student’s t-test for means and *X* ^2^ test for proportions. A propensity score was calculated for each participant using forward logistic regression [16], including all baseline covariables. Participants were divided into 5 strata based on quintiles of the propensity score. General linear modeling (GLM) was used to test the significance levels for the covariables between the study groups after adjustment for the quintiles of the propensity score (Table 1). Numbers and crude incidence rates per 1,000 person-years of outcomes by groups of HbA1c ≤58 mmol/mol or >58 mmol/mol are given in Table 2.

**Table 1:** Baseline characteristics in people with type 2 diabetes aged 25–90 years, by HbA1c ≤58 mmol/mol (≤7.5%) or >58 mmol/mol (>7.5%).

	Baseline HbA1c				Updated mean HbA1c			
	≤58(≤7.5%)	>58(>7.5%)	Pvalue^#^	Pvalue^*^	≤58(≤7.5%)	>58(>7.5%)	Pvalue^#^	Pvalue^*^
Number	12550	12926	–	–	12478	12998	–	–
HbA1c, %	6.9 (0.5)	8.5 (0.8)	–	–	6.7 (0.5)	8.5 (0.9)	–	–
Age, year	66.4 (12.0)	65.7 (11.1)	<0.001	0.175	66.8 (11.7)	65.3 (11.4)	<0.001	0.471
Men, N (%)	7167 (57.1)	7092 (54.9)	<0.001	0.634	7075 (56.7)	7184 (55.3)	0.022	0.447
Duration, year	7.2 (7.0)	10.8 (7.7)	<0.001	0.005	7.4 (7.1)	10.5 (7.7)	<0.001	0.059
BMI, kg/m^2^	28.2 (4.7)	28.6 (4.9)	<0.001	0.989	28.2 (4.7)	28.7 (4.9)	<0.001	0.466
Smokers, N (%)	1540 (12.3)	1800 (13.9)	<0.001	0.557	1482 (11.9)	1858 (14.3)	<0.001	0.804
Insulin, N (%)	3424 (27.3)	7177 (55.5)	<0.001	0.001	3382 (27.1)	7219 (55.5)	<0.001	<0.001

Data are given as means (SD) or numbers (frequencies %). ^#^ without propensity score * with stratification by quintiles of propensity score.

**Table 2:** Numbers and incidence rates of outcomes in people with type 2 diabetes aged 25–90 years, by HbA1c ≤58 mmol/mol (≤7.5%) or >58 mmol/mol (>7.5%).

			Baseline HbA1c				Updated mean HbA1c			
	All patients		≤58 mmol/mol		>58 mmol/mol		≤58 mmol/mol		>58 mmol/mol	
	N	Incidence	N	Incidence	N	Incidence	N	Incidence	N	Incidence
All cancer	3433	15.73	1727	15.95	1706	15.52	1731	16.10	1702	15.38
Gastrointestinal cancer	826	3.42	415	3.45	411	3.38	405	3.38	421	3.45
Kidney and urinary cancer	86	0.35	46	0.38	40	0.32	50	0.41	36	0.29
Respiratory cancer	86	0.35	46	0.38	40	0.32	43	0.35	43	0.35
Female genital cancer	183	1.67	86	1.64	97	1.73	82	1.55	101	1.82
Breast cancer	309	2.89	143	2.77	166	3.00	143	2.74	166	3.03
Prostate cancer	740	5.70	391	5.94	349	5.46	403	6.21	337	5.20

N: Number of outcomes. Incidence rate: numbers/1,000 person-years.

**Figure 2:**
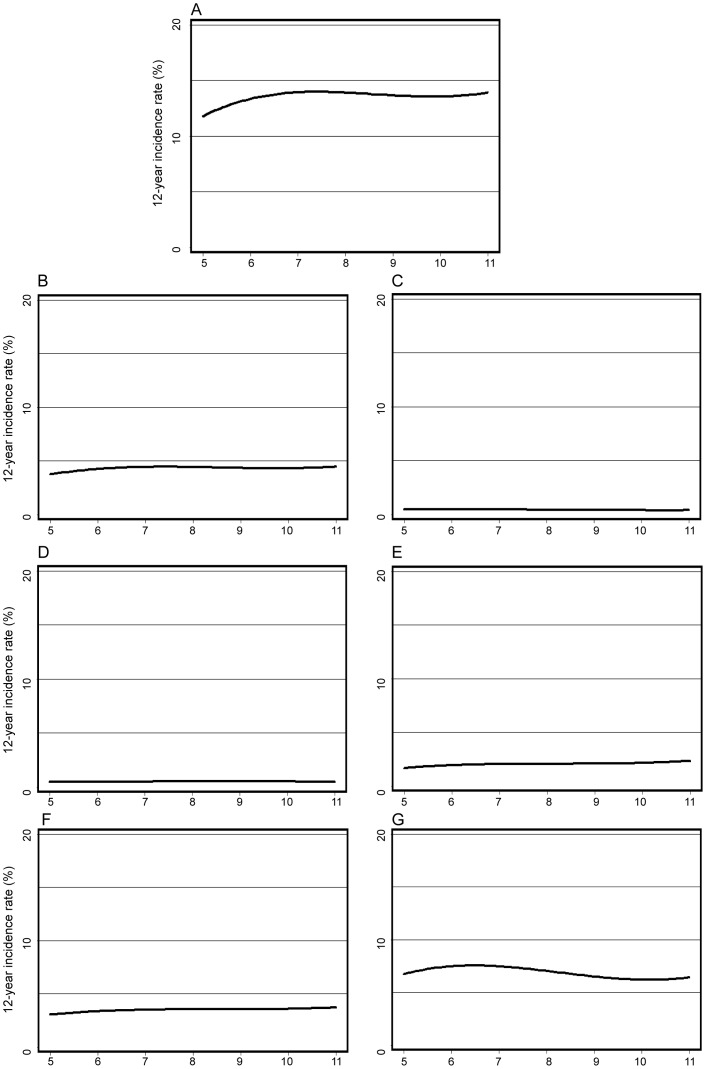
12-year incidence rate of cancer in a Cox regression model, fully adjusted as in model 2 in **table 3**. A. 12-year incidence of all cancer; B. 12-year incidence of gastrointestinal cancer; C. 12-year incidence of cancer in kidney and urinary organs; D. 12-year incidence of cancer in respiratory organs; E. 12-year incidence of cancer in female genital organs; F. 12-year incidence of breast cancer; G. 12-year incidence of prostate cancer.

Cox proportional hazard regression was used to estimate hazard ratios (HRs) with 95% confidence intervals (CI) for outcomes (Tables 3, 4 and 5). The follow-up time was used as the time scale [17]. The updated mean HbA1c value was treated as a strictly time-dependent variable in the Cox regression to evaluate glycemic exposure during follow-up, allowing for the use of a recent value of updated mean HbA1c at each specific time point in the modeling process. We used three different models for adjustment when comparing groups with HbA1c ≤58 or >58 mmol/mol (Table 3). Model 1 estimated crude hazard ratios. Model 2 adjusted for age, sex (except in sex-specific cancers), diabetes duration, smoking, and insulin treatment as covariates. Model 3 used stratification with quintiles of a propensity score including the same covariates as in Model 2. In addition, we estimated HR for outcomes with higher quartiles of baseline or updated mean HbA1c and the lowest quartile as reference, adjusting for covariates according to Model 2 (Tables 4–5). Finally, HR were estimated for outcomes per one %-unit increase in baseline HbA1c as continuous variable, adjusting according to Model 2 (Table 3). A Cox regression model was also used to estimate 12-year incidence rate of outcomes, in which model output was the 12-year rate for each participant, adjusted for covariates as given in model 2 (Figure 2).

**Table 3:** Hazard ratios (HR) and 95% confidence intervals (95% CI) ) for all cancer and specific cancers by baseline or updated mean HbA1c at Cox regression, in people with and type 2 diabetes followed for 12 years from 1997–99 to 2009.

	Baseline HbA1c	Baseline HbA1c (mmol/mol)				Updated mean HbA1c (mmol/mol)			
	Per 1%	≤58	>58			≤58	>58		
	increase		Model 1	Model 2	Model 3		Model 1	Model 2	Model 3
	HR (95% CI)		HR (95% CI)				HR (95% CI)		
All cancer	1.01(0.98–1.04)	Ref	0.99(0.92–1.06)	1.02(0.95–1.10)	1.02(0.95–1.09)	Ref	1.01(0.95–1.08)	1.04(0.97–1.12)	1.03(0.96–1.10)
Gastrointestinalcancer	1.00(0.94–1.06)	Ref	0.98(0.86–1.12)	1.03(0.89–1.20)	1.02(0.88–1.18)	Ref	1.02(0.89–1.17)	1.12(0.97–1.29)	1.09(0.94–1.26)
Kidney andurinary cancer	1.00(0.94–1.06)	Ref	0.86(0.56–1.32)	0.86(0.55–1.36)	0.87(0.55–1.37)	Ref	0.71(0.46–1.09)	0.70(0.44–1.10)	0.70(0.44–1.10)
Respiratorycancer	1.00(0.85–1.19)	Ref	0.87(0.57–1.32)	0.83(0.53–1.31)	0.84(0.53–1.32)	Ref	0.99(0.65–1.52)	1.00(0.64–1.57)	1.00(0.63–1.56)
Female genitalcancer	1.00(0.89–1.12)	Ref	1.06(0.79–1.41)	1.03(0.75–1.41)	1.06(0.77–1.46)	Ref	1.17(0.88–1.57)	1.18(0.86–1.61)	1.22(0.89–1.68)
Breast cancer	1.01(0.92–1.10)	Ref	1.08(0.87–1.36)	1.08(0.85–1.38)	1.09(0.86–1.39)	Ref	1.10(0.88–1.38)	1.12(0.88–1.43)	1.12(0.88–1.43)
Prostate cancer	1.00(0.94–1.06)	Ref	0.97(0.84–1.12)	1.04(0.89–1.21)	1.05(0.90–1.22)	Ref	0.92(0.80–1.07)	0.98(0.84–1.14)	0.96(0.82–1.11)

Model 1: Crude HR, without adjustment for covariates. Model 2: Adjustment for age, sex, diabetes duration, BMI, smoking, and insulin treatment as covariates. Model 3: Adjusted HR after stratification with a propensity score. Adjustment by stratification with quintiles of a propensity score including covariates as in Model 2.

**Table 4:** Cancer incidence rate (1/1,000 person-years) and hazard ratios by quartiles of baseline HbA1c in participants with type 2 diabetes.

	Quartile 1	Quartile 2	Quartile 3	Quartile 4
**All cancer**				
No. of cases	853	874	877	829
Incidence rate per 1,000 person-years	15.71	16.18	15.69	15.36
Hazard ratio (95% CI)	Reference	0.99 (0.90–1.09)	1.00 (0.91–1.11)	1.03 (0.93–1.14)
**Gastrointestinal cancer**				
No. of cases	195	220	218	193
Incidence rate per 1000 person-years	3.24	3.66	3.52	3.23
Hazard ratio (95% CI)	Reference	1.10 (0.91–1.34)	1.11 (0.91–1.36)	1.07 (0.86–1.32)
**Prostate cancer**				
No. of cases	205	186	182	167
Incidence rate per 1000 person-years	6.17	5.70	5.97	5.00
Hazard ratio (95% CI)	Reference	0.91 (0.75–1.11)	1.02 (0.83–1.26)	0.95 (0.76–1.18)
**Breast cancer**				
No. of cases	77	75	83	74
Incidence rate per 1000 person-years	2.73	2.88	3.10	2.87
Hazard ratio (95% CI)	Reference	1.02 (0.74–1.40)	1.12 (0.81–1.55)	1.02 (0.72–1.44)
**Female genital cancer**				
No. of cases	47	45	45	46
Incidence rate per 1000 person-years	1.64	1.70	1.65	1.77
Hazard ratio (95% CI)	Reference	0.99 (0.66–1.50)	0.98 (0.64–1.50)	0.99 (0.64–1.55)

Hazard ratios were adjusted for age, sex (except in sex-specific cancers), diabetes duration, smoking, and insulin treatment.

HbA1c quartile 1: <50 mmol/mol (<6.7%); quartile 2: 50–57 mmol/mol (6.7–7.4%); quartile 3: 58–68 mmol/mol (7.5–8.4%); quartile 4: >69 mmol/mol (≥8. 5%).

**Table 5:** Cancer incidence rate (1/1000 person-years) and hazard ratios by quartiles of updated mean HbA1c in participants with type 2 diabetes.

	Quartile 1	Quartile 2	Quartile 3	Quartile 4
**All cancer**				
No. of cases	906	846	838	843
Incidence rate per 1000 person-years	17.03	14.97	14.87	16.17
Hazard ratio (95% CI)	Reference	0.90 (0.82–0.99)	0.94 (0.85–1.04)	1.08 (0.98–1.20)
**Gastrointestinal cancer**				
No. of cases	207	202	221	196
Incidence rate per 1000 person-years	3.49	3.21	3.58	3.39
Hazard ratio (95% CI)	Reference	0.96 (0.79–1.16)	1.12 (0.92–1.36)	1.12 (0.91–1.38)
**Prostate cancer**				
No. of cases	219	185	176	160
Incidence rate per 1000 person-years	6.99	5.47	5.24	5.15
Hazard ratio (95% CI)	Reference	0.84 (0.69–1.02)	0.86 (0.70–1.06)	0.94 (0.76–1.17)
**Breast cancer**				
No. of cases	78	72	74	85
Incidence rate per 1000 person-years	2.94	2.64	2.66	3.36
Hazard ratio (95% CI)	Reference	0.88 (0.64–1.22)	0.92 (0.66–1.28)	1.15 (0.82–1.61)
**Female genital cancer**				
No. of cases	40	50	46	47
Incidence rate per 1000 person-years	1.49	1.80	1.64	1.83
Hazard ratio (95% CI)	Reference	1.17 (0.77–1.78)	1.08 (0.70–1.69)	1.18 (0.74–1.87)

Hazard ratios were adjusted for age, sex (except in sex-specific cancers), diabetes duration, smoking, and insulin treatment.

HbA1c quartile 1: <51 mmol/mol (6.8%); quartile 2: 51–57 mmol/mol (6.8–7.4%); quartile 3: 58–66 mmol/mol (7.5–8.2%); quartile 4: >67 mmol/mol (≥8.3%).

Proportional hazard assumption was tested with the Kolmogorov-type supremum test using re-sampling, and introducing the test of all time-dependent covariables simultaneously. Violations of the proportional hazards assumption were detected for age in the analysis of any type of cancer or prostate cancer, and this variable was therefore included as a strata variable (quartiles of age) in the Cox regression models. Similarly, violations were detected for BMI in the analysis of cancer of respiratory organs, and BMI quartiles were included in the model as a strata variable. In addition, we analyzed adjusted hazard ratios for incidence of all cancer and specific cancers by quartiles of baseline HbA1c values, as well as by quartiles of updated mean HbA1c values, using the lowest quartile 1 as reference (Tables 4–5). Interactions between HbA1c categories and all covariables were analyzed with maximum likelihood estimation, with no interaction detected. All statistical analyzes were performed using SAS (SAS Institute, US).

### Ethics

The data linking of national registers required for this study was approved by the Regional Ethics Review Board at the University of Gothenburg. All data analyzed were anonymous; therefore, informed consent for each individual was neither necessary according to Swedish legislation act 2003:460 concerning research on humans, nor is it possible when data is anonymous.

## Results

The study cohort was divided into two groups by the baseline HbA1c value 58 mmol/mol (7.5%), and into two groups by the updated mean HbA1c value 58 mmol/mol. The baseline characteristics of each group are given in Table 1.

Mean HbA1c was 6.6% and 8.4% in the groups with baseline HbA1c≤58 mmol/mol and >58 mmol/mol, and 6.7% and 8.5% in the groups with updated mean HbA1c ≤58 mmol/mol and >58 mmol/mol, respectively. The group with higher baseline HbA1c was significantly younger, had fewer men, longer diabetes duration, higher BMI, more smokers, and was more often treated with insulin at the start of follow-up. Similar differences were observed regarding the groups based on higher versus lower updated mean HbA1c. After stratification by quintiles of a propensity score, there were no significant differences in mean age, diabetes duration, and all differences except the insulin treatment disappeared.

Table 2 showed the numbers and incidence rates of incident cancers in total and by subgroups of baseline or mean updated HbA1c ≤58 mmol/mol and >58 mmol/mol. In total, we observed 3,433 cancers in the cohort, yielding an incidence rate of 15.73/1,000 person-years.

Table 3 presents HR for risk of cancer with patient groups of HbA1c >58 mmol/mol versus those with ≤58 mmol/mol, using three different models. There were no significant differences in risks of any cancer or specific cancer in groups of baseline HbA1c>58 mmol/mol compared to ≤58 mmol/mol, or in groups of updated mean HbA1c>58 mmol/mol compared to ≤58 mmol/mol.

Table 3 also shows HR for risk of cancer by one per cent unit increase in baseline HbA1c as a continuous variable. These HR were all non-significant for all cancer or cancer of specific types. Figures 2 A–G presents complementary splines of 12-year incidence rates of all cancer and cancer of specific types across the range of baseline HbA1c at a Cox model with adjustment as in Table 3.

In our additional analysis, we calculated adjusted hazard ratios for incidence of all cancer and specific cancers when we divided the cohort by quartiles of the baseline HbA1c values. The 25^th^, 50^th^ and 75^th^ percentiles of baseline HbA1c were 50 mmmol/mol (6.7%), 58 mmol/mol (7.5%) and 69 mmol/mol (8.5%). With the lowest quartile 1 as reference, no significant differences in risk of all cancer or cancer of gastrointestinal, prostate, breast or female genital organs were found in the higher quartiles 2–4 (table 4).

Additionally, we estimated adjusted hazard ratios for incidence of all cancer or specific cancers when we divided the cohort by quartiles of the updated mean HbA1c values. The 25^th^, 50^th^ and 75^th^ percentiles of updated mean HbA1c were 51 mmol/mol (6.8%), 58 mmol/mol (7.5%) and 67 mmol/mol (8.3%). This analysis showed a decreased risk of all cancer in quartile 2, hazard ratio 0.90 (0.82–0.99), while no significant differences in risk were seen in the highest quartiles 3 and 4, as compared with quartile 1. No significant differences in risk for cancer of gastrointestinal, prostate, breast or female genital organs were found in the higher quartiles 2–4 as compared with quartile 1 (table 5).

## Discussion

In this large-scale nationwide population-based cohort study, we did not observe associations between higher HbA1c as a marker of elevated blood glucose levels, i.e. poor glycemic control and incidences of all cancers or specific types of gastrointestinal, breast or prostate cancer, cancer in kidney and urinary organs, respiratory organs or female genital organs in patients with type 2 diabetes.

The rationale for using HbA1c = 58 mmol/mol (7.5%) as the cutoff point for dichotomized comparison was that it was the median HbA1c value. Furthermore, use of categorization by the median HbA1c allowed for comparisons between groups with a mean difference in HbA1c as high as 1.5–2%.

The unique features of our study is that the cohort only consisted of patients with type 2 diabetes, and that we used HbA1c which indicates the blood glucose level over the last 1–3 months. Thus, our study differs from some cohorts studies which used fasting or post-load blood glucose [18]–[20]. as well as from previous studies from Sweden, Korea and Austria, which were based on healthy survey data where the majority of cohort members were non-diabetics [18]–[20]. Although these studies had large sample size, the proportion of participants with diabetes was either unknown [20], or only 2–5% [18], [19]. The effect of fasting serum glucose on cancer risk in diabetic participants was not reported in these studies.

No increased or decreased risks of any cancer or specific types of cancer were found in participants with poor blood glucose as compared with good blood glucose control in patients with type 2 diabetes in our study. This finding is consistent with the results from meta-analyses of major trials data of the UKPDS, the ACCORD study and the VADT (Veterans Affairs Diabetes Trial) study, [9] and a study with the General Practice Research Database and secondary care data [21] also reported no association between intensified glycemic control and cancer risk. A recent report based on the ADVANCE study included 5,571 participants with intensive blood glucose control and 5,569 with standard control [7]. Both groups had a mean baseline HbA1c of 58 mmol/mol (7.5%) and at the end of follow-up the intensive control group had mean HbA1c 6.5% and the standard control group 7.2%. After a median follow-up of 5 years, no significant differences in any cancer risks between the two groups were observed.

Our results are not consistent with the Hongkong study of type 2 diabetes which was based on 973 new insulin users and 971 matched non-users of insulin [22]. This study found that HbA1c per percentage was associated with a 1.24-fold increase in cancer risk. However, follow-up duration was quite short, the mean follow-up being 3.01 years for insulin users and 0.70 years for nonusers. The outcome numbers were small, with 32 cancer cases in insulin users and 120 in non-users. Insulin users had significantly higher HbA1c values than non-users (8.1% vs 7.1%). The authors acknowledge that HbA1c was not collected systematically during follow-up.

Existing observational epidemiological data on associations between blood glucose and cancer risks have shown contradictory results, some based on healthy people or mixed groups with or without diabetes [10], [12], [23]–[27]. Increased HbA1c values were found to be related to an increased risk of gastric cancer in Japan, based on a cohort among which the majority had no diabetes [23]. No association between HbA1c level and risk of colorectal cancer was reported from studies based on women in the Nurses’ Health Study, the Women’s Health Study [10], [12], in patients with type 2 diabetes [24], or in studies based on European Prospective Investigation into Cancer and Nutrition [25], [26]. Similarly, no association between HbA1c level and risk of breast cancer was reported in apparently healthy women in the Women’s Health Study [28].

Diabetes has been reported to be associated with decreased risk of prostate cancer [29]. The reason for this remains unclear. Higher prostate-specific antigen (PSA) level is a marker of prostate cancer. The inverse association between HbA1c and PSA was reported in some studies [30], [31] but not all [32]. Two of the studies which found inverse associations, were cross-sectional studies [30], [31]. A two-year longitudinal study [32] of 5,917 Japanese men aged 50 and over found increased PSA with increased HbA1c level. However, a two-year follow-up is quite short for a cancer study.

A comparison between lower and higher quartiles of HbA1c for all cancer or specific cancers risk was additionally performed in this study, as quartiles of the HbA1c distribution may have higher statistical power than dichotomization by the median HbA1c value. A slightly decreased all cancer risk of borderline significance when comparing updated mean HbA1c quartile 2 with the quartile 1 could be neglected, as no effect on all cancer risk was found in the higher quartiles 3 and 4. Furthermore, no significant differences in risks for specific cancers were observed in quartiles 2–4 as compared with to quartile 1. Finally, analysing HbA1c continuously per 1 per cent unit increase showed no increased risk for all cancer or specific cancers (Table 3), as also demonstrated with splines of 12-year incidence rates of all cancer and specific cancers (Figure 2).

The main strengths of our study were the large sample size based on high quality registers, the long follow-up period with thorough follow-up, the complete information concerning baseline HbA1c levels and cancer outcomes, and the possibility of adjusting for relevant potential confounding factors. We could determine the temporal sequence of the casual relationship, if any, since our study has well documented time for HbA1c values, the diagnosis of diabetes and the studied cancers.

Our study has some limitations. Firstly, not all patients with diabetes in Sweden are registered in the National Diabetes Register. However, the selection for our cohort was not related to the study outcome – incident cancer. Thus, the risk of selection bias is minimal. Secondly, HbA1c measurement error might be a concern. We used both baseline and updated mean HbA1c as markers of glycemia. HbA1c is considered a stable indicator of the past 1–3 months’ blood glucose level. Also, the nationwide program to calibrate HbA1c levels and guidelines of reporting ensures high accuracy of HbA1c and reduces measurement errors. Thirdly, the use of different diabetes medications might be related to altered risks of incident cancer [14], [33]. Since the Prescribed Drug Register was initiated in Sweden on July 1^st^ 2005, we lack information on specific diabetes medication in this study with its baseline in 1997–1999. However, as we have information in the NDR on whether the patients use insulin, a variable indicating whether the patients were on insulin treatment or not at baseline was used as a covariate in the multivariate models. Fourthly, certain information was not available in our data at recruitment time, such as lipid values, markers of inflammation, comorbidities, and endogenous insulin levels. Lipid values were reported to increase cancer risk in type 2 diabetes [34]. Since hyperglycemia might induce abnormal lipids [35], lipids are intermediate factor in the causal pathway between hyperglycemia and cancer. Thus, lipids are not confounders in our study [36].

In summary, there were no significant differences in incidences of all cancer or cancer of specific types between groups with baseline HbA1c≤58 mmol/mol (7.5%) and HbA1c>58 mmol/mol (7.5%), or between groups with updated mean HbA1c≤58 mmol/mol (7.5%) and HbA1c>58 mmol/mol (7.5%), in patients with type 2 diabetes.
